# Correlations between different plaque indexes and bleeding on probing: A concurrent validity study

**DOI:** 10.4317/jced.60039

**Published:** 2023-01-01

**Authors:** Ana-Paula Carvalho, Marcela-Faria Moura, Fernando-Oliveira Costa, Luís-Otávio-Miranda Cota

**Affiliations:** 1 School of Dentistry, Department of Dental Clinics, Oral Pathology, and Oral Surgery, Periodontology Division, Federal University of Minas Gerais, Belo Horizonte, Minas Gerais, Brazil

## Abstract

**Background:**

It is stated that plaque indexes emphasizing interproximal areas or gingival margins are important when periodontal inflammation is the main focus. This cross-sectional study aimed to evaluate the concurrent validity between the following indexes: Greene & Vermillion (GV), Quigley & Hein modified by Turesky (QHT), Silness & Löe (SL), Ainamo & Bay (AB), O’Leary (OL), Deinzer (DZ), and bleeding on probing (BOP).

**Material and Methods:**

A sample comprising 183 individuals underwent complete periodontal examination and were divided according their periodontal status. BOP was expressed in percentages of affected sites for the entire mouth and for lingual, buccal and interproximal surfaces. Spearman correlations were calculated for each index and BOP at each area.

**Results:**

Overall, correlations were moderate between all indexes and BOP at all areas, except for the OL index that showed weak correlations. The concurrent validity increased for the gingival health group, reaching strong correlations between the AB, GV, DZ indexes and BOP at the entire mouth. In the gingivitis and periodontitis groups, the concurrent validity decreased, with most correlations reaching weak or non-significant values.

**Conclusions:**

In cross-sectional evaluations, the GV, QHT, SL, AB and DZ indexes showed good validity concurrent with BOP, regardless of their specific characteristics.

** Key words:**Bleeding on probing, concurrent validity, correlation study, periodontal diseases, dental plaque index.

## Introduction

Periodontal diseases are complex bacterial infections involving an intricate interaction between subgingival microbiota, host immune responses and modifying factors (1). The dysbiotic polymicrobial biofilm and the periodontal inflammation are critical to understanding its pathobiology. Both play a major role in the aetiology and pathogenesis and they reinforce each other within the context of the continuum of health, gingivitis and periodontitis (2,3). Dental biofilm is undoubtedly the principal cause of the initial inflammatory lesion (1).

A proper periodontal examination is a critically important step in clinical practice to reach proper diagnosis and develop a treatment plan, as well as in periodontal research to provide a reliable and comprehensive data collection (4,5). Current methods applied for periodontal examination basically remain very similar to those from early decades. Overall approaches comprise recording clinical signs of periodontal health, inflammation and destruction, assessments of etiological factors and oral hygiene performance, together with consistent medical and dental histories (6,7).

Gingival bleeding is an early and accurate sign of inflammation. Its measurement mainly consists of qualitative or semi-quantitative indexes based on the tendency of the gingiva bleeding upon mechanical stimulation (6,8). The presence of bleeding on probing in a dichotomous manner is considered to be the simplest way to assess periodontal inflammation (4).

Given that dental biofilm plays an essential role in initiating periodontal inflammation, and also that plaque control is the foundation of periodontal health (9,10), assessments of dental plaque should be a regular component of periodontal examination for routine clinical practices and research purposes (7,10). In this sense, many plaque-scoring methods are available. They are mostly based on a subjective assessment of the amount of tooth surface covered by plaque, ordinal ratings of plaque extension, or the presence/absence of plaque at specific sites (11-13).

Different plaque indexes have been proposed and mainly focused on oral hygiene skills, but also on periodontal issues. They have demonstrated good quality, discriminatory power and reliability (14-17). It has been advocated that more sensitive indexes, those discriminating between high and low plaque scores and those emphasizing the interproximal areas and/or the gingival margins are necessary when periodontal inflammation is the main focus (11,12).

Consequently, it would be of great interest to broaden the understanding of how different plaque indexes correlates to periodontal inflammation. Concurrent validity is an approach of criterion validity that estimates the amount of agreement between two different assessments. It focuses on the power of a test to predict outcomes on another test. This could guide professionals and researches in the choice of a plaque index according to their specific goals in the periodontal practice and research. Hence, the aim of the present study was to evaluate the concurrent validity between different plaque indexes and bleeding on probing.

## Material and Methods

-Study design and sampling strategy

The present cross-sectional study comprised a convenience sample of male and female individuals, 18-65 years old (43.21±13.24), selected in the Periodontology Clinic at the School of Dentistry from the Federal University of Minas Gerais, Belo Horizonte – Brazil. Sample size calculation was based on the expected correlation coefficient, 0.80 study power and 0.05 statistical significance (18,19). In this manner, for coefficients varying from 0.20, 0.30, 0.40 and 0.50, a minimum sample size varying from 194, 85, 47 and 29 individuals were respectively determined to be necessary.

During the period of data collection (August/2019 to December/2019) approximately 320 individuals sought dental care at the clinic and were determined eligible. Individuals were invited to participate according to their accessibility and availability during the dental care routine. Exclusion and inclusion criteria were then applied to each individual. Participants had to have a minimum of 20 natural teeth with 12 interproximal areas (20) and no contraindication for periodontal examination. Individuals with a history of systemic diseases, pregnant or lactating women, smokers/former smokers (21), those with any orthodontic appliances, removable protheses, extensive prosthetic rehabilitations or extensive active caries lesions were excluded. Exclusion was also applied to individuals using any drugs that could influence periodontal health and those who have used antibiotics and anti-inflammatory drugs within the last 3 months prior to study entry (22). Using this approach, final sample comprised 183 individuals.

The present study was approved by the Research Ethics Committee of the Federal University of Minas Gerais – Brazil (protocol #16932919.4.0000.5149) and was conducted in accordance with the Helsinki Declaration. Participants were made aware about the research objectives and signed an informed consent.

-Data collection

Individuals underwent oral examination and dental plaque recording. In a specific dental chart, after using a disclosing agent (basic fuchsin solution), the distribution of plaque accumulation (PA) was schematically represented for all dental surfaces of all present teeth.

Subsequently, this representative graphic chart was used to retrieve the score values of the following plaque indexes:

1) GV – Greene & Vermillion (23): buccal and lingual surfaces of all present teeth were given scores of 0= no debris present, 1= soft debris covering not more than 1/3 of the tooth surface, 2= soft debris covering more than 1/3 but not more than 2/3 of the tooth surface, 3= soft debris covering more than 2/3 of the tooth surface. The higher score attained to any of the buccal and lingual surfaces in each right, anterior and left segments of each upper and lower arches were summed and divided by the number of selected surfaces and the number of evaluated segments;

2) SL – Sillness & Löe (17): the first right molar, the right lateral incisor and the first left premolar in the upper arch as the left first molar, the left lateral incisor and the right first premolar in the lower arch were evaluated. Each buccal, lingual, mesial and distal surface was given scores of 0= no plaque, 1= a film of plaque adhering to the free gingival margin and adjacent area of the tooth only seen by using the probe, 2= moderate accumulation of soft deposits on the tooth or gingival margin which can be seen by a naked eye, 3= abundance of soft matter on the tooth and gingival margin. Scores of each examined surface were summed and divided by the number of examined surfaces;

3) QHT – Quigley & Hein modified by Turesky *et al*. (24): each lingual and buccal surface of all present teeth was given scores of 0= no plaque, 1= separate flecks of plaque at the cervical margin of the tooth, 2= thin continuous band of plaque (up to 1mm) at the cervical margin of the tooth, 3= a band of plaque wider than 1mm but covering less than 1/3 of the tooth crown, 4= plaque covering at least 1/3 but less than 2/3 of the crown of the tooth, 5= plaque covering 2/3 or more of the tooth crown. All scores were summed and divided by the number of examined surfaces;

4) OL – O’Leary *et al*. (25): each buccal, lingual, mesial and distal surface was recorded as positive if they presented soft accumulations at the dentogingival junction. The number of plaque-containing surfaces were divided by the number of examined surfaces;

5) AB – Ainamo & Bay (26): each buccal, lingual, mesial and distal surface was recorded as positive if they were covered with clearly visible plaque. The frequency of positive surfaces was expressed in percentages in relation to the total number of examined surfaces;

6) DZ – Deinzer *et al*. (12): the presence of plaque was assessed at the lingual and buccal gingival margin divided in 4 equal sections (distal, cervico-distal, cervico-mesial, mesial). The percentage of all sections scoring positive in relation to all examined sections were attained.

The main characteristics of the plaque indexes used in this study is presented in Table 1.

**Table 1:**
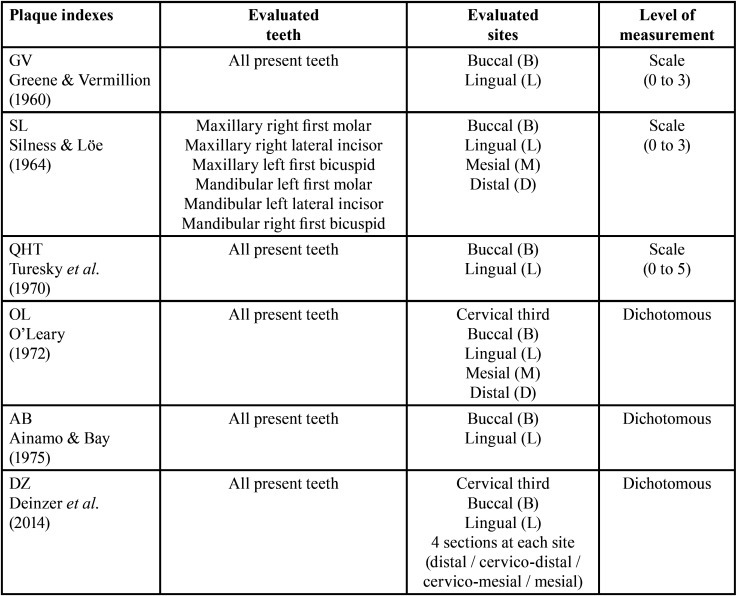
Main characteristics of the plaque indexes used in the study.

-Periodontal examination and periodontal status definition

All participants have also undergone a full-mouth periodontal examination performed with a manual periodontal probe (PCPUNC-15, Hu-Friedy, Chicago, IL, USA). Probing depth (PD), clinical attachment level (CAL), and bleeding on probing (BOP) were recorded from all present teeth, except from third molars, at mesial, distal, buccal and lingual sites. BOP was recorded in a dichotomous manner (presence/absence).

All examinations were performed by one single trained periodontist (A.P.C). A sample of 5 individuals were examined and re-examined within 1 week to determine intra-examiner agreement for PD and CAL. Kappa values >0.88 and intraclass correlation coefficients >0.90 were attained.

Participants were then classified according to their periodontal status – a) gingival health: no/minimal BOP (score <10% assessed as the proportion of bleeding sites) (8,9); b) gingivitis: BOP score ≥10% and PD ≤3mm (8); c) periodontitis: detecTable interdental CAL (not ascribed to non-periodontal causes) at ≥2 non-adjacent teeth or buccal CAL ≥3mm with PD >3mm at ≥2 teeth (those diagnosed with at least periodontitis stage I) (27).

-Statistical analysis

Sample was characterized regarding percentage of sites with plaque accumulation (PA) and bleeding on probing (BOP) at the entire mouth (PAE and BOPE), as well as at buccal (PAB and BOPB), lingual (PAL and BOPL) and interproximal (PAI and BOPI) sites, respectively. Correlations between plaque index and BOPE, BOPB, BOPL, BOPI were evaluated in the total sample, as well as in the study groups. Magnitude of correlations were classified as: negligible (0.00–0.10), weak (0.10–0.39), moderate (0.40–0.69), strong (0.70–0.89) and very strong (0.90–1.00) (28,29). All analyses were performed using statistical software (SPSS 17.0, Statistical Package for Social Sciences for Windows, SPSS Inc., Chicago, IL.). Results were considered significant if *p*<0.05.

## Results

Sample comprised 183 individuals, being 43 diagnosed with gingival health, 44 with gingivitis and 96 with periodontitis. Percentage of sites with PA and BOP are presented in Table 2. BOP was lower at buccal sites and higher at interproximal sites. BOP markedly increased from the gingival health to the gingivitis and periodontitis groups. PAI, PAB and PAL were not expressively different. PA and plaque index scores also markedly increased from the gingival group to the gingivitis and periodontitis groups. Overall, differences were found not so expressive between the gingivitis and periodontitis groups.

**Table 2:**
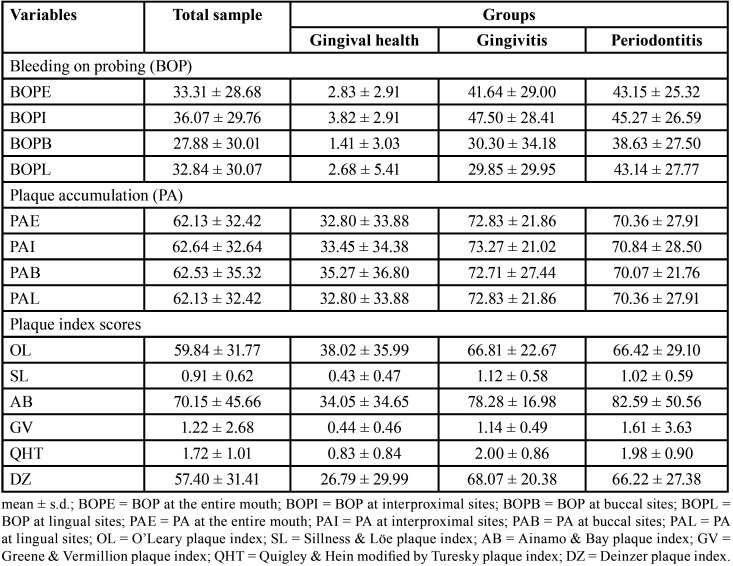
Characterization of the sample in relation to bleeding on probing (BOP), plaque accumulation (PA) and plaque index scores.

Table 3 shows correlations between plaque indexes and BOP. Overall, correlations were determined to be moderate for all plaque indexes, except from OL index. When considering the total sample, correlations varied from 0.20 to 0.57 for BOPE. OL index presented weak correlations with BOPE, BOPI, BOPB, BOPL. SL, AB, GV, QHT and DZ indexes presented moderate correlations with BOPE, BOPI, BOPB, BOPL, varying from 0.42 to 0.57. QHT index showed higher correlation coefficients, but were still considered moderate when compared to the other plaque indexes. Lower correlation coefficients were observed for BOPB.

**Table 3:**
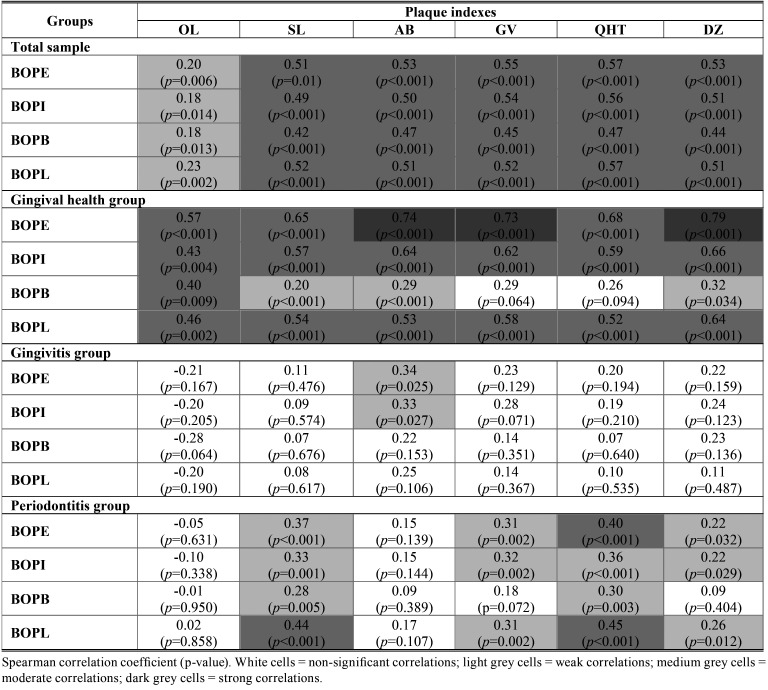
Correlations between plaque index scores and bleeding on probing (BOP).

When considering the gingival health group, higher correlations were obtained, varying from 0.57 to 0.79 for BOPE. It is noteworthy that AB, GV and DZ indexes presented strong correlations, DZ being the highest (0.79). Correlations between PA and BOPB were lower, being classified as weak for SL, AB and DZ indexes and non-significant for GV and QHT indexes. OL index presented coefficients with less variation, all determined to be moderate for BOPE, BOPI, BOPB and BOPL.

When considering the gingivitis group, correlations were mostly non-significant, except from AB index that showed weak correlations with BOPE (0.34) and BOPI (0.33). When considering the periodontitis group, correlations were non-significant for OL and AB indexes. Moderate correlations were observed between SL and BOPL (0.44), QHT and BOPE (0.40), and QHT and BOPL (0.45). Overall, correlations between plaque indexes and BOP on both groups presented the highest variations.

## Discussion

Overall, SL, AB, GV, QHT and DZ indexes showed good concurrent validity with BOP assessed in the entire mouth (BOPE), considering the total sample. Independently from the main differences in plaque assessment of these indexes, they presented correlations varying from 0.51 to 0.57, all being determined to be significantly moderate. Some indexes may be easier to access and demand less time or logistics while other may be more time consuming. Thus, findings demonstrate that in situations as epidemiological studies with large samples, choosing a plaque index that demands less time and logistics for its application, may be appropriate and benefit data collection. It was previously stated that simple indexes might be of similar significance to more detailed and graded indexes (30-32).

Due to its scalar evaluation, it was stated that QHT index can be an appropriate tool to understand PA patterns (13). Plaque quantification indexes are generally accepted for their advantage of being more sensitive in the evaluation of clinical variables since dental plaque is quantified at each site on an incremental scale. However, they may be time consuming to administer. On the other hand, binary or dichotomous indexes are easier to use and their validity relies on the fact that the presence or absence of a clinical variable such as plaque or gingival bleeding which are important, not necessarily its quantification (31).

Indeed, good correlations among plaque indexes with different characteristics were demonstrated (11,12,32,33). Good convergent validity between QHT and DZ index was demonstrated under different conditions (12). DZ index evaluates the presence/absence of PA in 4 sections of gingival margins at the buccal and lingual surfaces. It was advocated that this assessment of PA at the gingival margins may have similar meanings to those presented through more complex indexes and provide nearly the same information as scoring its distribution over the whole tooth surface (12).

The QHT index showed the highest correlation with BOPE. This index was developed with the purpose of evaluating oral hygiene. It scores PA from 0 to 5, according to the continuity and extent of dental plaque covering the tooth crown, assessed in the buccal and lingual surfaces at the entire mouth (24). It is one of the most used indexes in product-testing and oral hygiene studies and demonstrated good correlation with quantitative plaque assessments such as weight (32) and image analysis (33). It was also demonstrated that the QHT index presented good concurrent validity with BOP under different conditions. Moreover, QHT index showed good convergent validity with DZ index that presented similar concurrent and predictive validity for gingival bleeding (12). It is interesting to observe that indexes focusing on PA at gingival margins, such as DZ index, or those considering PA at interproximal areas did not present higher correlations with BOPE or BOPI.

When individuals in the gingival health group were evaluated separately, correlations improved and reached higher levels for the DZ, AB and GV indexes when evaluating BOPE. The DZ index showed the highest correlation with BOPE. In relation to the other sites, a similar behaviour of the total sample was observed with moderate concurrent validity, except for BOPB. In the gingivitis and periodontitis groups, correlation patterns were inconsistent. Overall, concurrent validity was weak and / or non-significant. This behaviour could be related to the presence of several factors that can interfere or potentiate overall gingival inflammation (9,27,34). Variations in gingival bleeding may be the response to other factors besides the presence of PA (35). Correlations between BOP and plaque indexes at vestibular sites were lower, what could be explained by the easiness of cleaning this area (36).

Different associations between PA and BOP were previously demonstrated in different teeth and sites and they were weaker in molars and lower anterior teeth, as well as at buccal sites. It is noteworthy that in this present study, PAE, PAI, PAB and PAL presented similar values while BOPB showed comparatively lower values and BOPI comparatively higher values. However, it was indicated that individuals with a strong association between PA and BOP and individuals with no or even with negative associations had similar mean percentages of plaque and BOP (22). Variations in correlation patterns were explained by the study type, hormonal changes, individual characteristics and oral hygiene interventions (12,17,22,37,38). BOP may be associated with site-specific factors as well as patient-related factors (9,35). Nevertheless, it was demonstrated that on a population level, a considerable correlation between gingival index and plaque index is achieved (22,39).

Although there may be different relationships between PA and BOP at the site level, it seems accepTable to assume that higher and appropriate correlations exist between PA and extent of BOP at the individual level (ecological correlation) (22,38). Summary measures from different plaque indexes for the individual, although numerically different due to index specific characteristics, seems to define a PA profile that has an overall satisfactory concurrent validity with BOP.

The selection of an appropriate plaque index will depend upon: goals of the study in which it is to be utilized; sample size; study design; cross-sectional or longitudinal evaluations; duration of the study and association with the outcomes of interest (13,15,40). In specific clinical situations, clinical trials and epidemiological studies aiming at evaluating the distribution of clinical variables in more detail, complex quantification indexes may be necessary (31). On the other hand, in studies with larger samples and more demanding logistics, a plaque index with easier application could be eligible. In general, plaque indexes are used in routine dental examination to describe the pattern of PA and patient oral hygiene. In dental research, they are widely used in clinical trials to evaluate products for oral hygiene and oral hygiene regimens. Moreover, in observational studies focusing on periodontal conditions, plaque indexes are used to characterize individuals and they may reflect the exposure to the primary causal factor of the periodontal inflammation itself.

Particularly in cross sectional evaluations, plaque index assessment may be subjected to some temporal ambiguity. Plaque index values in a one single examination at a specific point in time may not reflect patterns of past exposure to PA. However, there is a mutual interdependence between plaque amount and gingival inflammation, especially in a steady state. Although there are factors involved in inter and intra-individual variations, gingival changes objectively reflected by gingival bleeding occur in the face of PA. It is generally accepted that a correlation exists between the amount of PA and the extent gingival inflammation (38). The present study demonstrated that different plaque indexes showed significant moderate concurrent validity with periodontal inflammation, independently of their PA quantification systems. Therefore, it can be assumed that there is no plaque index with a particular characteristic that can, in general, better correlates with BOP.

It is important that the conduction of periodontal probing exam must be performed correctly. It is recommended to carry out a “gentle probing” of periodontal tissues, as an excess of pressure can lead to false positive results (26,34). In the present study, all examinations were performed by the same trained examiner in a very systematic manner. This systematization of data collection is essential for the results to be standardized, and training is a critical step on indexes recording and its subjective aspects. In addition, BOP was recorded in an objective dichotomous manner, and it was previously demonstrated that bleeding on marginal probing and bleeding on sulcus probing are significantly correlated at individual and at site-level evaluations (38).

A convenience sample with particular characteristics was investigated in the present study. Consequently, external validity must be interpreted with caution. The study focused on stablishing the concurrent validity of plaque indexes comprising different quantification systems with BOP. This could help choosing the index that best fits the objectives, logistics, clinical situation and time of periodontal evaluation, whether in clinical practice or in dental research, when concurrent validity with BOP is intended. Further studies with larger samples, as well as samples comprising specific clinical conditions, are necessary to better evaluate the concurrent validity of plaque indexes and gingival bleeding.

## Conclusions

It was concluded that the evaluated plaque indexes presented moderate concurrent validity with gingival bleeding when evaluated in the total sample, regardless of their specific teeth/sites under evaluation or quantification systems. Hence, the choice of a plaque index when focusing on the concurrent validity with gingival bleeding should take the logistics of the examination, the study sample and design into consideration.
